# Case–control study exploring the short-term association of bronchiolitis with high blood pressure and hypertension in hospitalized children

**DOI:** 10.1186/s40885-022-00214-5

**Published:** 2022-10-01

**Authors:** Sophia Giang, Andrew J. Padovani, Lavjay Butani

**Affiliations:** Department of Pediatrics, University of California Davis, Room 348, 2516 Stockton Boulevard, Sacramento, CA 95817 USA

**Keywords:** Hypertension, Blood pressure, Bronchiolitis, Hospitalization, Child

## Abstract

**Background:**

Unlike in adults, there are limited pediatric data exploring the association between acute respiratory illnesses and blood pressure abnormalities. The aim of our study was to explore the association of bronchiolitis, a common respiratory illness, with elevated blood pressure in hospitalized children.

**Methods:**

In this single center retrospective case–control study, we evaluated the association between bronchiolitis and elevated blood pressure and hypertension in hospitalized children, compared to a control group admitted with nonrespiratory conditions, using multivariate regression analyses. Standard published normative data on pediatric blood pressure were used to classify children in various blood pressure categories.

**Results:**

A high prevalence of elevated blood pressure (16%) and hypertension (60%) was noted among children with bronchiolitis; this was not statistically different from the control group (18% for elevated blood pressure; 57% for hypertension; *P*-values, 0.71 and 0.53, respectively). On multivariate regression analyses, only length of stay was associated with hypertension. No patient with blood pressure abnormalities received antihypertensives nor were any nephrology consults documented.

**Conclusions:**

A high prevalence of blood pressure abnormalities, without documentation of their recognition, was noted in hospitalized children regardless of diagnosis, pointing to the need for more data on outcomes-driven significance of pediatric inpatient blood pressure measurements.

## Background

The literature provides evidence of a theoretical link between the lungs and blood pressure (BP). Studies in adult patients have shown a strong comorbidity between bronchial asthma, a respiratory illness, and hypertension (HTN) [1]. Strengthening this link are genomic studies that have elucidated potentially similar pathophysiologic causes for these two conditions, with both sharing genes involved in cytokine signaling, protection against oxidative stress, histone encodement, platelet function, and activity of adrenoreceptors—a known target of drugs for both HTN and asthma [2]. Additionally, investigations into the pathophysiology of two other infectious respiratory conditions, respiratory syncytial virus (RSV) and the novel coronavirus SARS-CoV-2 infection, have yielded a potential causal mechanism between pulmonary inflammation and elevated BP via the renin-angiotensin system (RAS). The respiratory diseases caused by RSV and SARS-CoV-2 are associated with downregulation of angiotensin-converting enzyme (ACE) 2, which leads to a subsequent rise in the key RAS component, angiotensin II [3, 4].

While there is literature to support an association between respiratory illnesses and HTN in adults, there have been no studies investigating the association of acute respiratory illnesses with the development of BP abnormalities, either in adults or in children. Based on our personal observations of frequent BP abnormalities in children admitted to our hospital with bronchiolitis and the frequent questions related to these abnormalities to the pediatric nephrology team by treating health care providers, we conducted our current study to explore this association. Bronchiolitis is the leading cause of hospitalization in US infants, accounting for 16% of all hospital admissions in patients in the first year of life [5, 6]. Rhinovirus and RSV-induced wheezing illnesses, including bronchiolitis, during infancy are also associated with the future development of asthma in school-age children, with a significantly higher odds ratio for rhinovirus-mediated disease [7]. As admission for bronchiolitis may be the first manifestation of an underlying inflammatory lung disease, children with bronchiolitis present a potential opportunity for investigating the link between respiratory illnesses and HTN since these children would otherwise not be screened for HTN until 3 years of age [8].

Given the high volume of patients hospitalized for bronchiolitis each year and the dearth of scientific investigation into the association between bronchiolitis and HTN, we conducted a retrospective case–control study with the primary aim of determining the prevalence of BP abnormalities in children admitted for bronchiolitis; in order to explore whether BP abnormalities in these children were related to pulmonary inflammation or a consequence of the stress response related to the febrile illness and/or hospitalization, we compared BP data in children admitted for other common nonrespiratory conditions. We hypothesized that hospitalized children with bronchiolitis would have a higher prevalence of BP abnormalities, HTN and elevated BP (HBP), compared to children hospitalized for other acute illnesses. Our secondary objective was to identify clinical and patient demographic factors associated with HBP and HTN and to characterize the management and follow-up of BP abnormalities in the outpatient setting.

## Methods

### Ethics approval

The study was approved by the Institutional Review Board of the University of California Davis. An exemption for the need for written informed consent was granted by the Institutional Review Board of the University of California Davis due to the study's retrospective nature. All methods were performed in accordance with the relevant guidelines and regulations.

### Subjects

Children < 3 years of age admitted to either the pediatric wards or intensive care unit (ICU) services between January 2017 and January 2019 with a discharge diagnosis of bronchiolitis were included in this study. An age and sex-matched control group, consisting of children admitted to the aforementioned services with one of the following medical conditions: skin and soft tissue infection, urinary tract infection, or seizure was included for comparison. These diagnoses were chosen for the control group as they are common reasons for admission in children < 3 years, do not affect respiratory status, and may be distressing in terms of pain and discomfort. The intent of this control group was to explore whether there was a specific link between bronchiolitis and BP abnormalities that may be attributed to the disease process itself rather than the agitation associated with an inpatient hospitalization or the discomfort of having respiratory distress unrelated to the underlying diagnosis (i.e., white coat HTN).

### Exclusion criteria

Subjects needed to have at least 1 day of a minimum of two “legitimate” BP readings recorded in the flowsheet. Measurements were deemed “legitimate” if there was no nursing documentation that the patient was agitated or moving during the reading, or that the BP was measured on a lower extremity. Notably, those measurements not specifying from which extremity they were obtained were assumed to be taken from an upper extremity, as is standard practice in our hospital, and included in the analysis. Patients were excluded from the study if they had previously diagnosed HTN or were on antihypertensive medications prior to admission, or if they were intubated at any point during the hospitalization, as many sedation medications have vasoactive properties that can affect BP. Other exclusion criteria included a diagnosis of bronchopulmonary dysplasia, presence of acute kidney injury, electrolyte abnormalities, a comorbid acute process overlapping with one of the other inclusion diagnoses, and absence of a recorded height in children as this is needed for determination of BP percentiles.

### Study design

This was a single center retrospective case–control study. HBP and HTN were defined as at least one inpatient day of either averaged diastolic BP (DBP) or averaged systolic BP (SBP) readings above the 90th and 95th percentile, respectively, determined by using age-appropriate BP charts. Standard published normative pediatric reference charts were used based on the age of the child [8–10]. The most recent clinical practice guideline for screening of BP, published in 2017, was used for children ≥ 1 year of age [8]. Since normative data on younger children was not a part of these guidelines, the report of the second task force on BP control in children was used for infants 1 month to 1 year of age [9], and data developed by Zubrow et al. [10] was used for infants < 44 weeks of corrected gestational age. In the cohort admitted for seizures, only those BP readings that were obtained after the child had regained normal sensorium were included in the study data. BP measurements were taken by trained pediatric nursing personnel using a validated oscillometric measuring device (Dinamap; Critikon Company LLC, FL, USA). Legitimate BP measurements were transcribed from flow sheets, and SBP and DBP were individually averaged over each inpatient day. These daily averages were then assigned percentiles using the appropriate reference charts. To quantify the severity of HTN, when present, the HTN index was calculated by dividing daily averaged SBP and DBP values by the respective reference 95th percentile values [11]. Data relating to patient demographics and clinical management that are known to, or could possibly, influence BP were collected: age, sex, body mass index (BMI), use of systemic corticosteroids or albuterol, length of stay, history of prematurity, and severity of illness as determined by wards/ICU admission status. Additional data abstracted included mention in the medical records of recognition of BP abnormalities, inpatient treatment of or outpatient referral for BP abnormalities, and outpatient BP measurements for patients who were followed in our health-system. Postdischarge outpatient BP measurements were obtained by reviewing all clinical encounters taking place within 3 months of hospital admission and accessible through the electronic health record. The primary outcome measure was the prevalence of BP abnormalities during the hospital stay—HBP, HTN, and the composite of both HBP and HTN. The secondary outcome measure was the severity of BP abnormalities, as quantified by the HTN index.

### Statistical approach

Sample size calculations were based on our assumption that children admitted with bronchiolitis would have a tenfold higher prevalence of HTN (10%), compared to the control groups (1%). To achieve a power of 80% to detect this difference and an α of 0.05, we needed 100 children in each of the two groups. Multivariate logistic regression analyses were performed using Stata MP ver. 16 (Stata Corp., College Station, TX, USA). Regressions were estimated separately for the three different primary outcome measures: HTN, HBP, and the composite of HBP or HTN. Model specification was tested by performing linktests, comparing Bayesian information criteria, and computing the area under the model’s receiver operating characteristic curve. HTN and HBP were defined as the dependent variable outcomes of the following independent variables: sex, corrected gestational age, normative chart used, BMI > 90th percentile, level of care, length of stay, a diagnosis of bronchiolitis, prematurity, and use of albuterol or steroids as a part of inpatient treatment. Comparisons of HTN indices for bronchiolitis and control groups was performed using the Student t-test.

## Results

### Demographics

**Fig. 1:**
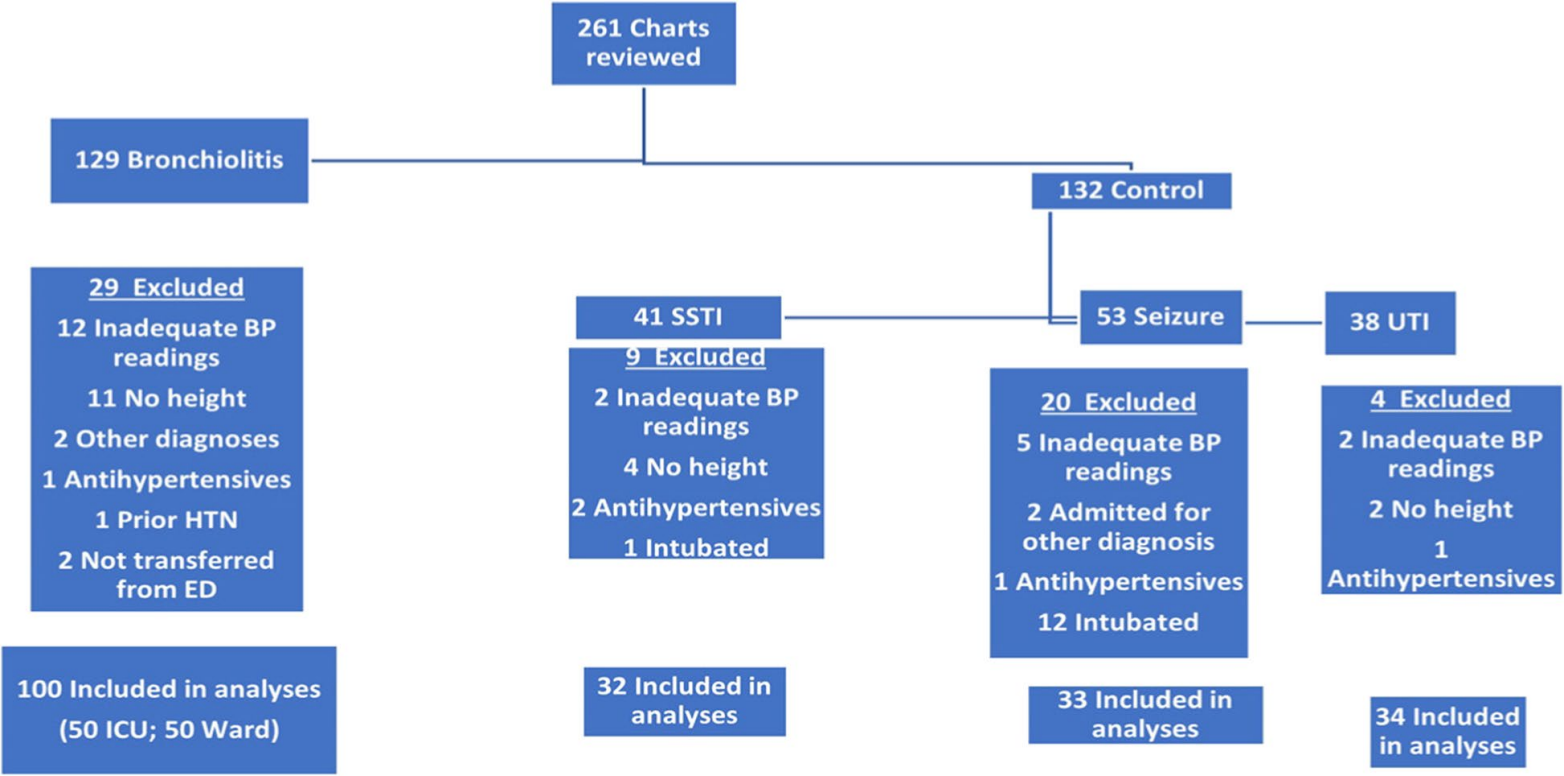
Schematic flow chart of reviewed medical records and included study subjects. BP, blood pressure; HTN, hypertension; ED, emergency department; SSTI, skin and soft tissue infection; UTI, urinary tract infection; ICU, intensive care unit

During the study period, there were 370 children admitted to the hospital with a discharge diagnosis of bronchiolitis. Charts were reviewed in reverse chronology until the target sample size was reached. A total of 129 patient charts were reviewed to reach the target of 100 patients meeting inclusion criteria. The same method was used for the control group with a target of 99 total controls consisting of an equal distribution from each diagnosis category. The most common reasons for exclusion were inadequate BP readings and omission of recorded height; of patients with a diagnosis of seizures, a high proportion was excluded due to their intubation status (Fig. 1). Figure 1 is a schematic displaying data on the numbers of screened medical records and reasons for exclusion in each diagnosis group.

Table 1 depicts demographic characteristics of the various study subjects. The bronchiolitis and control populations were similar in average corrected gestational age and mean length of stay. Males and premature infants were overrepresented in the bronchiolitis group as compared to the controls. Among bronchiolitis patients, those requiring wards versus ICU levels of care were also comparable in demographic factors.

**Table 1:** Demographic characteristics of study subjects

Characteristic	Bronchiolitis (*n* = 100)	Control (*n* = 99)	*P*-value
Mean corrected age (mo)	10.8	11.4	> 0.05
Ward	9.6	-	
ICU	12.1	-	
Female sex (%)	39	56	0.01
Ward	40	-	
ICU	35	-	
Mean length of stay (day)	3.2	2.7	> 0.05
Ward	2.7	-	
ICU	3.7	-	
BMI > 90th percentile (%)	26	36	> 0.05
Ward	18	-	
ICU	34	-	
Premature (< 34 wk, %)	19	5	0.002
Ward	24	-	
ICU	16	-	

*ICU* Intensive care unit, *BMI* body mass index

### Blood pressure data

The prevalence of HBP and HTN in patients with bronchiolitis was 14% and 62%, respectively, as compared to the prevalence of 18% and 57% in the combined control group. A diagnosis of bronchiolitis was not found to be significantly associated with HBP or HTN. The odds ratio (OR) of observing HTN in patients with bronchiolitis as compared to those without was 0.63 (95% confidence interval [CI], 0.15–2.69), with a corresponding *P*-value of 0.53. For HBP, the OR was 0.17 (CI, 1.95e^–05^–1,547; *P* = 0.71). The OR of observing either HBP or HTN with bronchiolitis was 0.27 (CI, 0.06–1.21; *P* = 0.09). To determine whether the severity of respiratory illness is associated with HTN, we compared the prevalence of HTN between patients with bronchiolitis requiring ward versus ICU levels of care. As compared to children admitted to the wards, those who initially went to the ICU were older (although not significantly so) and had a greater length of stay. Both groups demonstrated a male predominance. The prevalence of HTN was 48% versus 76% for patients admitted to the wards and ICU, respectively. When comparing all bronchiolitis and control patients, ICU status was not found to be significantly associated with HTN (*P* = 0.95). The SBP and DBP HTN indices were not significantly different between the patients with bronchiolitis and the control group (data not shown).

The multivariate logistic regression models also accounted for several other patient and clinical management factors that were identified as possible contributors to HTN. As seen in Table 2, the only factor found to statistically correlate with HTN was length of stay. Use of steroids, use of albuterol, prematurity, corrected gestational age, and sex did not significantly influence the odds of HBP or HTN.

**Table 2:** Multivariate regression analyses for three different outcomes: HTN, HBP, and HTN or HBP

Variable	HTN	HBP	HTN or HBP
Bronchiolitis	0.627	0.174	0.270
* P*-value	0.530	0.706	0.087
95% CI	0.146–2.693	0.001–1,547	0.060–1.210
Intensive care unit stay	1.061	2.555	1.226
* P*-value	0.947	0.542	0.828
95% CI	0.185–6.075	0.125–52.23	0.195–7.707
Corrected gestational age	1.062	1.222	1.116
* P*-value	0.394	0.258	0.158
95% CI	0.925–1.220	0.863–1.729	0.958–1.300
Male sex	0.758	1.883	1.255
* P*-value	0.536	0.512	0.669
95% CI	0.316–1.820	0.284–12.47	0.444–3.548
Overweight or obese	1.103	0.736	1.303
* P*-value	0.848	0.769	0.667
95% CI	0.406–2.994	0.096–5.674	0.390–4.357
Albuterol use	1.333	3.43	2.556
* P*-value	0.694	0.799	0.324
95% CI	0.318–5.586	0.001–46,093	0.396–16.49
Steroid use	0.812	0.375	0.887
* P*-value	0.758	0.817	0.902
95% CI	0.216–3.056	0.001–1,506	0.133–5.923
Prematurity	8.157	-	3.025
* P*-value	0.295	-	0.623
95% CI	0.160–415.5	-	0.037–248.8
Length of stay	1.367	0.824	1.287
* P*-value	0.036	0.587	0.175
95% CI	1.020–1.832	0.409–1.659	0.894–1.853

All coefficients are reported as odds ratios. Bootstrapped *p*-values and 95% Confidence Intervals are also provided

*HTN* Hypertension, *HBP* Elevated blood pressure, *CI* Confidence interval

### Follow-up

For the 199 inpatient encounters included in the analysis, there were no pediatric nephrology consultations placed and very rare documentation addressing abnormal BP values. There were no instances of pharmacotherapy being initiated for the treatment of HTN. Of the 160 patients identified as having HBP or HTN, only 19 (12%) received at least one outpatient BP measurement within 3 months of hospital discharge. Sixteen of these 19 patients (68%) had outpatient measurements also in the HBP or HTN range with no documented normotensive recordings. Within the bronchiolitis group, 20% of those categorized as hypertensive during their hospitalization received outpatient BP measurement following discharge. Of those, 83% never had a normotensive reading; 75% of this group had received steroids. However, all but one measurement was done > 1 week after discharge after which any steroid burst would have been completed (median time to follow-up BP, 41 days; range, 3–90 days).

## Discussion

In our study, we evaluated a possible association between bronchiolitis in hospitalized children and BP abnormalities. We found that, compared to control subjects admitted for nonrespiratory illnesses, those with bronchiolitis did not have a higher prevalence of HBP or HTN after controlling for clinical and demographic factors. Among children admitted for bronchiolitis, those with a greater severity of illness were likewise no more likely to be hypertensive than their lesser sick counterparts. The only significant correlations with HTN were greater length of stay.

The prevalence of HBP and HTN in our study was surprising to us and is strikingly high. The explanation for this is likely multifactorial and could include agitation resulting from the process of obtaining BP measurements and underlying illness (akin to white coat HTN effect seen in the outpatient setting), BP measurements obtained on the lower extremity or using an inappropriate technique, or inaccurate height recordings making assessments of BP abnormalities erroneous. Our definition of HTN in this study is likely prone to overestimating the prevalence and necessarily differs from the definition conventionally used for diagnosis, which is based on the outpatient setting (auscultatory-confirmed BP readings ≥ 95th percentile on three different visits) [8].

Although these high prevalence rates likely do not reflect the actual prevalence of HTN in this population, they do underscore several challenges that pediatric providers face in detecting clinically significant BP elevations in the inpatient setting. Despite increasing investigations into the incidence, diagnosis, and treatment of inpatient HTN, we have yet to reach a consensus definition and guidelines for monitoring and management of BP abnormalities in this population. Presently, the best estimate for the prevalence of inpatient HTN based upon the literature is somewhere between 1 and 25% [12]. The wide range reflects the variable definitions and the populations included in the studies. The more conservative estimates come from studies where the diagnosis was established retrospectively, based on a billing code diagnosis of HTN at discharge; the more liberal estimates are from studies using single emergency room admission BP measurements [13, 14]. Studies suggest that critical illness is a risk factor for acutely elevated BP; Ehrmann et al. [15] found the prevalence of HTN to be 25% among pediatric ICU patients when defining HTN as at least three SBP and/or DBP readings above the 99th percentile over 1 day. Most importantly, this study correlated HTN using this definition to clinical outcomes such as increased odds of acute kidney injury and increased length of stay [15].

While our study hypothesis was not confirmed, i.e., that bronchiolitis is associated with BP abnormalities in hospitalized children, we cannot entirely refute that an association between acute respiratory illnesses and BP abnormalities exists. This stems from the aforementioned issues pertaining to absence of normative BP data and the lack of an evidence base behind BP screening in hospitalized children, who are a very different population compared to children seen in the ambulatory setting; differences that arise, as mentioned previously, from the anxiety, stress and agitation of the underlying illness and the need for hospitalization. The very limited documented postdischarge follow-up on BP abnormalities that were noted during the hospital stay is further cause for concern. Our observations open up new questions and areas of potential investigation that are worthy of attention, not only for their implications on patient care but also due to the cost involved with interventions and provision of nursing care (involved in BP measurements) and the additional stress, both physical and psychological, imposed on children and their families with frequent measurement of vital signs and BP determinations.

Limitations of this study include the fact that it was conducted in a single center and its retrospective design, which precludes standardization of nursing documentation pertaining to BP records. Thus, abnormal BPs were not confirmed using manual BP measurement, which is the recommended protocol as oscillometric readings are known to overestimate both DBP and SBP [8]. Moreover, documentation of agitation, movement, and site of BP measurement were likely inconsistent. It also cannot be guaranteed that all cases of medical interventions such as systemic steroid use are accounted for in those patients transferred from outside hospitals; inclusion of outside hospital events is dependent upon the physician authoring the history taking and physical examination. Lastly, it is possible that there is a true difference in BP between patients admitted for bronchiolitis as opposed to a nonrespiratory illness, that was masked by BP elevations from whitecoat HTN or agitation. Again, further studies on clinically significant BP values and trends among hospitalized patients will be instrumental in improving management of these patients and avoiding adverse outcomes potentially tied to acute or sustained BP abnormalities. Until then, we posit the following as actionable measures to achieve better detection and interpretation of abnormal BPs among hospitalized children: (1) improved and more detailed documentation of BP, (2) electronic health record (EHR) clinical decision-making tools to draw attention to BP abnormalities, and (3) protocols to follow up abnormal BP. BP documentation should include mandatory fields such as patient position (e.g., supine, sitting), patient state (including the presence of pain, agitation, or movement), and site of BP measurement. Studies looking at implementation of BP-related clinical decision-making support (CDS) linked to the EHR show that such systems significantly improve provider detection of HTN, although the majority of these were performed on outpatient populations. In one study, use of a hard-stop and prompt to obtain a manual recording after entry of a BP value > 90th percentile increased provider detection of abnormal BP encounters by 29.5% [16]. The teen BP study evaluated efficacy of a more involved CDS that prompted for additional BP measurements and order sets depending on recorded BP values; its use increased recognition of incidental HTN by 33.6% [17]. With regards to follow-up protocols, each abnormal BP should be followed by a manual recording that can be prompted by the EHR. Additionally, discharge summaries should document whether a patient was persistently hypertensive through the course of hospitalization and suggest outpatient BP follow-up for those patients with comorbidities associated with HTN.

## Conclusions

Our study highlights the profound need for additional studies to establish evidence-based and clinical outcome-driven guidelines for defining BP and categorizing BP abnormalities in the inpatient setting. The issue of diagnosing white coat hypertension in this population (related to the stress, anxiety and/or pain related to the hospitalization process or underlying illness) remains challenging and worthy of investigation. Whether there is a role for ambulatory BP monitoring in hospitalized patients (who are, by the very nature of their hospitalization, not in their normal and stress environment until after they are discharged, well and back in a familiar home environment) and the interpretation of the data obtained through such means, are additional questions to consider.

## Data Availability

The datasets used and/or analyzed during the current study are available from the corresponding author on reasonable request.

## Abbreviations

ACE: Angiotensin-converting enzyme
BMI: Body mass index
BP: Blood pressure
CDS: Clinical decision-making support
CI: Confidence interval
DBP: Diastolic blood pressure
ED: Emergency department
EHR: Electronic health record
HBP: Elevated blood pressure
HTN: Hypertension
ICU: Intensive care unit
OR: Odds ratio
RAS: Renin-angiotensin system
RSV: Respiratory syncytial virus
SBP: Systolic blood pressure
SSTI: Skin and soft tissue infection
UTI: Urinary tract infection
